# Medical Items and News of Interest to the Physician

**Published:** 1870-10

**Authors:** 


### Medical Items and News,

### Of Interest to the Profession.

CULLED FROM THE STANDARD MEDICAL JOURNALS OF THE WORLD.

NOTE.—These formulæ are intended only for the regular practitioner of medicine, and should be employed only by him, or under his immediate supervision.

**Ergot as a Hæmostatic.**

Dr. CURRAN, (*Med. Press and Circ.*), is favorably impressed with the action of ergot of rye, in hæmorragic cases of typhoid fever. In a case published by him, one full dose of ergot entirely averted the hæmorrhage.

**Epilepsy.**

GEORGE JOHNSON, M. D., F. R. S., Physician to King's College Hospital, advocates chloroform in connection with bromide of potassium in this affection. Chloroform wards off a threatened fit, and cuts short a violent and prolonged paroxysm.

**Brandy before Chloroform.**

JAMES SHAW, A. A. Surg. U. S. A. (*Med. and Surg. Reporter.*), writes as follows: "In all cases of surgical operations, I give a little brandy and water some fifteen or twenty minutes before administering chloroform, as a matter of safety in keeping up the heart's action."

**Neuralgia.**

Mix together one part of hydrochlorate of morphia and thirty parts of flexible collodion.

This is to be applied to the seat of neuralgia pains by means of a camel's hair brush. Should pain occur periodically, larger or smaller doses of the sulphate or the valerianate of quinine are to be given besides.—*L'Union Medicale.*

**Epithelial Cancer.**

Dr. MAGIN relates, (*Gazz. delle Cliniche*), three cases of epithelial cancer of the face cured by the external application, several times a day, of a solution of chloride of lime, 1 part to 15 of water; and internally a solution containing about 7 grains in 180 parts of water.

**Unguent for Prurigo.**

Dr. CHARVET, (*Bull. Gen. de Therap.*), after an experience of twenty years, believes that the following ointment is an infallible cure for prurigo: Axunge, simple or camphorated, sixty parts; citrine ointment, three parts; mix. A small portion should be spread over the affected surface.

**Syphilitic Headache.**

Dr. EDWARD H. McLEAN, (*Lancet,*) finds that the simple headache met with almost daily, in ordinary practice, is one of the sequelæ of syphilis. For this headache we have in the iodide of potassium a remedy which is a veritable specific. This remedy, in five grain doses, three times daily, will ordinarily cause the trouble to disappear within a few days.

**Dyspepsia and Oxaluria.**

Dr. H. S. THORNE, of Chicago, (*Mich. Univ. Med. Jour.*), gave the subjoined prescription to a man æt. 45, afflicted with dyspepsia and oxaluria: Permanganate of potassa in grain doses, to be made into pills with bread, three to be taken every day for ten days. A few days afterwards he returned to state that he was cured.

**Hydrate of Chloral in Cancer.**

Mr. WEEDEN COOKE, surgeon to the Cancer Hospital, London, (*Med. Press and Circ.*), is in the habit of administering hydrate of chloral in the different varieties of cancer, for the purpose of entirely relieving the patient from pain. As a night-draught he has found 20 grs. sufficient. But when pain is persistent the 10 gr. dose, three times daily, gives the greatest satisfaction. Squire's method of administration is recommended, viz., the addition of syrup of tolu and peppermint water.

**Use of Quinia Hypodermically.**

E. PAUL SALE, M, D., of Aberdeen, Miss., reports in the *N. O. Jour. Medicine*, the following case of tetanus produced by the hypodermic injection of quinia: the patient, aged 19, mother of twins, æt. three months, was suffering from *malarial coma*, the result of a tertain intermittent fever of two months' duration. Desiring to rapidly quininize her, he administered in the arm, by hypodermic injection, quinia, gr. vj., of an ethereal solution. Four days afterward the arm was much tumefied, hot, and very painful to the touch, when the syringe was inserted.—Twelve days from this injection, Dr. Sale was summoned to the patient's bedside to prescribe for trismus with opisthotonos; but she succumbed after receiving the best of treatment. The chief point developed by this case is this: It shows the deleterious effects which frequently follow the use of quinia hypodermically. This is the fourth case out of ten in which he has had cause to regret resorting to this method of medication, on account of violent inflammation which has been the sequence.

**Enlarged Tonsils.**

We have been using for the past year, with much satisfaction, a paste prepared by mixing equal parts of caustic lime and soda. Procure an ounce of unslacked lime, pulverise it, and mix it thoroughly with the soda. Bottle it and when it is desired for use, place a small quantity on a piece of glass and mix it to the consistance of a stiff paste by adding alcohol. Then apply to the tonsil with a pine-stick spatula. Allow the patient to gargle the throat in twenty minutes, with cold water. *Ed. Bistoury.*

---

**Tar Water.**

M. MAGNES LAHENS suggests a method of preparing this water which is more expeditious and convenient than the plan commonly followed. He mixes the tar with sand, previously washed and dried, throws the mixture into a percolator, and shakes the instrument gently to secure proper adjustment of the mixture. Water is then poured on, the first part of the filtrate is rejected, and the latter portion is kept for use. He uses ½ oz. tar, 26 oz. of sand; to obtain two pints of the medicated water, which corresponds in strength with that of the codex.—*Journal de Pharm. et de Chimie.*

---

**Vomiting in Phthisis.**

A very common form of vomiting in phthisis is that which occurs during the evacuation of a large cavity. Various means have been employ- to mitigate these symptoms without material benefit. The subjoined formula is recommended by A. P. DUTCHER, M. D., (*Cin. Med. Reper*:)

℞

Acidi carboliæ, (cryst.), gr. iv;

Glycerinæ,

Tr. cinnamon, ââ ℥ j. M.

Sig:—A teaspoonful every four hours.

---

**Sulphate of Iron in Suppuration.**

A child burned all over the body, was recently brought to the Children's Hospital of Lausanne. The suppuration from its wounds were so abundant that the ward in which he was lodged became absolutely uninhabitable. M. JOEL then placed him in a bath containing two handsful of sulphate of iron. The cessation of pain was almost immediate; after repeating this bath twice a day, for fifteen or twenty minutes at a time, the suppuration moderated, the fetid odor disappeared, and the little sufferer recovered rapidly.—*Boston Jour. Chem.*

**Diabetes.**

Dr. GUYOT DANEZ recommends the citrate of soda in doses of 60 or 120 grains *per diem*, as an excellent remedy for diabetes. He says it is converted into a carbonate, which passes into the circulation without causing derangement; that the glucose disappears from the urine, and that it may be used as a substitute for salt, so that bread and other amylaceous food may be permitted in the diet.

---

**Surgeons for the Prussian Army.**

Since the opening of the war, twenty-five German Surgeons have sailed from New York to join the Prussian Army. COUNT VON THIELE, Prussian Foreign Secretary, states that the authorities at Berlin desire only those Surgeons who speak the German Language.

Surgeons who desire to obtain service in either the Prussian or French armies, should address the ministers of those nations, at Washington.

---

**How to Empty the Stomach.**

The *Deutsches Archiv* has published a paper by Dr. Jürgensen, of Kiel, which fully corroborates all that has been said as to the efficiency of a syphon to replace the stomach pump. The stomach may be readily emptied and as readily washed out by this simple mode. A sound has only to be placed in the stomach and its upper end attached to the short arm of a glass syphon by india-rubber tubing. The patient must then cough or retch, and the stomach, if full, will rapidly be emptied. Dr. Jürgensen employs this mode of washing out the stomach regularly with water, or with acid or alkaline liquids, in cases of gastric disease.

---

**Habitual Constipation.**

Prof. J. M. DA COSTA, of Philadelphia, prescribes as follows:

℞.

Podophyllin, gr. 1-20;
Extracti belladonnæ, gr. 1-20;
Capsici, gr. 1-4;
Pulveris rhei, gr. j. M.

For one pill. To be taken three times a day.

ANOTHER.

℞.

Confectio sennæ, ℥ j;

Potassæ bitartratis, ʒ ij;

Sulphuris præcipitatati,

Ferri subcarbonatis, ââ ʒ j;

Mellis despumati, q. s.

Ft. electuary. One teaspoonful after meals.

*Prof. John Forsyth Meigs.*

**Styptic Paper.**

A mode for carrying about ferric chloride as a ready styptic has been invented in Paris, which consists in dipping paper in a decoction of 1 lb. of benzoin, 1 lb. of alum in 4 gallons of water, which has been kept boiling for four hours, with renewal and skimming. The paper is left in the filtered solution for some time, until saturated; it is then dried, and painted over with a solution (neutral) of chloride of iron; this is then dried, folded, and wrapped in an impervious cover.—*Med. Archives.*

---

**Elixir of Chloroform.**

The following *Elixir Chloroform* is recommended by the Phamaceutical Association at Washington, D. C.

℞
Chloroform,
Tinct. opii,
Tinct. camph.,
Ip. ammon. ar., âã ℥ j ss;
Oil of Cinnamon, ♏xx;
Brandy, ℥. ij. M
Sig: Half a fluid drachm, more or less.

---

**Correction.**

In the July number of the BISTOURY, at page 139, occurs a formula taken from the *Cin. Med. Journal*, for Diptheria. Contrary to our habit, we entrusted the proof reading to another person, and a very important error occurred in this formula. We inclosed little printed notices in every BISTOURY, calling attention to the error, but for fear they may have been overlooked we here give the correction and formula as it should be:

℞

Tr, ferri chloridi, ʒ ij; potassiæ chloratis, ʒ ij; morphiæ muriatis, gr. ij; acidi muratici dil., ʒ ij; aquæ destillatæ, ℥ ij; syrup, ℥ ij; M. sig:—A teaspoonful every second or third hour.

---

**Rheumatic Fever.**

J. RUSSELL REYNOLDS, M. D., F. R. S., London, called attention to this agent (Perchloride of Iron) at the last meeting of the British Medical Association. The marked effects of tincture of percholoride of iron in such diseases as erysipelas and diptheroid sore throat had induced Dr. REYNOLDS to try it in acute rheumatism—which agreed with the others in coming under the class of spreading inflamatory affections. He had given it in eight cases, with such success as would justify a further trial. Having given brief histories of the eight cases, he directed attention to certain points: 1. The relief of the joint affections was definate, uniform and speedy. In four cases it was removed in one day; and the longest period of suffering after the commencement of the treatment was five days. 2. Excluding one fatal case with cerebral symptoms, and another where there was intercurrent pneumonia, the temperature became normal between the second and seventh days; the mean duration of pyrexia being a little less than five days and a half. 3. Excluding again the two exceptional cases already mentioned; the total duration of rheumatic fever from the outset, varied from seven to fifteen days, giving a mean of ten and a half days. 4. The earlier the iron was given, the shorter was the duration of the disease. No headache or other symptom of discomfort was produced by the iron.—*Boston Jour. Chem.*

---

**Typo-Cerebral Fever.**

A. GIVEN, M. D., Louisville, Ky., (*Chicago Med. Examiner*), in his able paper on typhoid fever, treats the *cerebral type* on general principles, but early directs attention to the delerium or head symptoms; for without sleep the patient will die, and that speedily. He has found nothing so beneficial in controlling the delerium and promoting sleep as the following:

℞

Chloroform, ʒ ij;

Pulvis acaciæ, Sacchra. alba, } âã ʒ iij;

Tr. Opii Deodor., ʒ ij;

Bromide Potassa, ʒ iv;

Aqua Menthæ, ℥ ij. M.

Sig: Give a teaspoonful every two hours, until the patient is quiet. If the head be hot, bathe it frequently in cold water. If the bowels do not move regularly, the enema of warm water and salt may be used from time to time.

---

**New Method of Transfusion of Blood.**

A physician of St. Petersburg, reports a new method of transfusion of blood through the *La Sante Publique.* He employs the blood of the cutaneous capillaries, rejecting the use of defibrinated blood and venous blood. For this purpose he employs a little apparatus, composed of a very thin cupping glass; a scarificator with nineteen small blades arranged in five rows, and exposed by pressure upon a button; a small pneumatic pump situated at the superior and lateral portion of the apparatus; a glass transfusion tube hermetically connected at the same apparatus, and capable of containing five ounces of blood, the tube terminating in a small trocar for opening the vein. The cupping glass and tube are enveloped in a jacket of caoutchouc, which, filled with water at 35° C., prevents the coagulation of the blood. The cupping-glass being placed upon the back of the individual from whom the blood is to be taken, and the tube, well closed, falling perpendicular below, it is kept in position with the left hand, while the right hand produces the vacuum by working the piston. As soon as the individual experiences a painful sensation, a stroke of the palm of the hand upon the button of the scarificator, plunges the blades into the skin and the blood flows. The tube being filled, the stopcock near the trocar is opened, and the trocar being plunged into the median cephalic or basilic vein of the invalid, the blood enters by force of gravity into the vessel. A turn of the stopcock interrupts the operation. A graduated scale upon the glass of the tube indicates at each instant the quantity of blood injected.

---

### Hydrate of Chloral in Delirium Tremens.

Dr. Stivers of the San Francisco City Hospital reports several cases from which we select the following one as a sample of them all: Case I.—E. W., female, aged 38, native of Ireland. Admitted April 13, at 9½, A. M., suffering from a severe attack of delirium tremens. The nurse was obliged to place her in a straight-jacket to have any control whatever of her. I ordered:

℞ Chloral hydrate, ʒ j;

Syrp. pruni virg.,

Aqua, āā ℥ j. M.

Sig. One half to be taken at once and the remainder in two hours, if no sleep.

The first dose was given at 10 A. M., the second at 12 M. No sleep, but patient rather more quiet. Repeated the same prescription, and gave one half at 2 P. M. Patient sleeping soundly at 2½ P. M., and continued in that condition until 9 P. M., when she awoke perfectly free from delirium; and to use her own words, she felt "pretty well—only a horrible headache." Took about a pint of beef tea. At 12 M. the remainder of the hydrate was given—in all, 2 dr. Directly afterwards she fell asleep, and so continued until morning, when she was discharged.

---

### Doses for Hypodermic Use.

We extract the following carefully prepared formulæ for the preparation of hypodermic medicines, from the *Cin. Med. Repostory*:

℞
Aconitine, gr. j;
Aqua destillatæ purissimæ, f ʒ ss.

Misce et fiat solutio. Dose: *minimum* ♏ ij. = 1-120 gr.; maximum, ♏ jv = 1-60 gr.

Atropine (*Atropia*, off.)—℞ Atropiæ sulphatis, gr. j; Aquæ destillatæ pur. f. ʒ ss. Misce et fiat solutio.

Dose:—*minimum*, ♏ ij = 1-120 gr.; *maximum* ♏ iv. = 1-48 gr. Adapted to pain in pelvic vicera.

Caffeine, Theine.—℞. Caffeinæ, gr. ij.; aquæ destil. bullientis, ♏ lxxx. Misce et fiat solutio. Dose: *minimum* ♏ 20 = ½ gr.; *maximum*, ♏ 40 = 1 gr. For neuralgia and alcoholic insomnia.

Strychina (*Strychina off.*)—℞ Strychinæ sulphatis, gr. ij; aquæ destil. bullientis, ʒ j. Misce et fiat solutio. Dose: *minimum*, ♏ ij = 1-120 gr.; *maximum*, ♏ iv gr. = 1-60 gr. In gastralgia and neuralgia of the heart.

Morphine (*Morphia, off.*)—℞ Morphiæ acetatis, gr. v.; acetici, ♏ j.; aqua destill. bull. f ʒ j. Misce et fiat solutio. Dose: *minimum*, ♏ i = 1-12 gr.; *maximum*, ♏ vi = ½ gr. Ten drachms to a drachm can be dissolved by use of glycerine. Especially useful in neuralgic pains and delerium tremens.

Nicotine (*Nicotina.*)—℞ Nicotinæ, gr. j; aquæ des. ℞ puris. f. ℥ ss. Misce et fiat solutio. Dose: *minimum*, ♏ ij = 1-120 gr.; *maximum*, ♏ iv = 1-60 gr. Useful in tetanus.

Digitaline (*Digitalia, off.*)—℞ Digitaliæ, gr. j; Aqua dest. puris. f. ℥ ss. Misce et fiat solutio. Dose: *minimum*, ♏ ij = 1-120 gr.; *maximum*, ♏ iv = 1-60 gr. Good in febrile conditions.

Calabar Bean (*Physostigma veneosum.*—℞—Extract. physostigmatos venosi alcoholici, gr. ij; aquæ destil. pur. ʒ ij. Glycerine, ℥ j. Misceet fiat solutio. Dose: *minimum*, ♏ 20 = 1-15 gr. *maximum*, ♏ 40 = ⅙ gr. In tetanus.

**Treatment of Pneumonia.**

The *American Practitioner* contains an article from Dr. J. HALL, from which we make the following extracts :—" In the first or congestive stage of pneumonia in plethoric subjects, in healthy, non-malarial districts, blood-letting is a valuable therapeutic agent; but in the second stage, and in malarial regions, as a rule, it is hazardous. I have not practised blood-letting nor used tartar-emetic in the treatment of pneumonia for ten years, because we have other therapeutical agents that answer better, and are attended with none of the dangers incident to the use of these agents. Veratrum viride and digitalis are the remedies upon which I rely to control the undue action of the heart and arteries. In the early stage of the disease, in robust subjects, after moving the bowels with a mercurial purgative. I give Norwood's tincture of veratrum in four drop doses, with ten grains of nitrate of potash, and from a fourth to a third of a grain of morphia, every four hours, increasing the tincture two drops every dose until the pulse is reduced to sixty five a minute, or nausea and vomiting occurs. I then reduce it to four drops, and continue it until the active stage of the disease has passed by ; then I stop the veratrum, but continue the nitrate of potash, with from one to two grains opium and half a grain of ipicac. ; and where there is a tendency to asthenia I add two grains of quinia to each dose. Under this treatment, frank, uncomplicated cases of pneumonia have seldom failed to terminate by resolution in from six to twelve days. But in some cases it has failed, and the fever has continued, with derangement of all the secretions, and complete hepatization of a portion of the lung tissue. In such cases I have derived great benefit from the use of hyd., digitalis, and opium, with free vesication over the diseased lung. I usually give one grain of Hyd. Chl. Mit, with two grains of digitalis and one of opium, every four hours, and continue until resolution is established, or the constitutional effects of the mercury are manifested. I then discontinue the hyd. and add two grains of quinia to each dose and continue until the febrile excitement is controlled.

The local treatment of pneumonia is of considerable importance, especially in pleuro-pneumonia, and all other cases attended by much pain. I generally rely upon cupping and sinapisms ; if they fail to afford relief, I inject a solution of morphia under the skin, over the sea of pain ; from a fourth to a half grain is sufficient. The inhalation of chloroform is also efficacious in such cases, and not only relieves the pain, but mitigates the fever, and I am inclined to think facilitates resolution of the inflammation. Warm applications are valuable in all cases. and should always be used when more active measures are not deemed necessary. In the advanced stage of this disease, when resolution is tardy, and effusion of serum takes place in the chest. I resort to free vesication. I have applied the tincture of iodine with very good effects in mild cases, and especially in the pneumonias of children—painting the entire walls of the chest two or three times a day. I have also used a liniment, in the cases of children and delicate females, composed of olive oil, turpentine, and amoina. * * * * * * * * Ventilation of the sick chamber should be thorough, and the temperature should be uniform at about 65° Fah.

